# Comparison of Transnasal Humidified Rapid-Insufflation Ventilatory Exchange and Facemasks in Preoxygenation: A Systematic Review and Meta-Analysis

**DOI:** 10.1155/2022/9858820

**Published:** 2022-07-13

**Authors:** Yongkai Li, Jianzhong Yang

**Affiliations:** Emergency Trauma Center, The First Affiliated Hospital of Xinjiang Medical University, Urumqi, China

## Abstract

**Background:**

Transnasal Humidified Rapid-Insufflation Ventilatory Exchange (THRIVE) has received increasing attention and application as an effective noninvasive mode of ventilation in the treatment of clinical anesthesia and critically ill patients. The conclusions reached in clinical studies of THRIVE and facemask oxygenation are still controversial, and the main objective of this systematic review is to determine the advantages of THRIVE over facemask oxygenation in intensive care units, respiratory medicine, and perioperative preoxygenation.

**Methods:**

PubMed, Embase, Web of Science, and Cochrane Library have search restrictions. The search library was full of English language articles from the first publication to 15 July 2021. Eligible randomized controlled study designs were included. 245 records were screened, and 5 studies met the inclusion criteria, enrolling a total of 235 patients.

**Results:**

Studying the THRIVE group compared to the facemask group, three studies analyzing intubation time showed that there is no difference in the effect of THRIVE and facemasks (MD -1.22, 95% CI -7.23 to 4.78, and *P* = 0.69 > 0.05). Three studies analyzing apnea showed that there was no difference between the two groups (SMD 1, 95% CI -0.76 to 2.76, and *P* = 0.27 > 0.05). Three studies analyzing PaO_2_ after preoxygenation showed that THRIVE is more effective than facemasks (MD 72.58, 95% CI 31.25 to 113.90, *Z* = 3.44, and *P* < 0.001). Two studies analyzing oxygen saturation SpO_2_ after successful intubation showed that there was no difference in the effectiveness (MD 0.09, 95% CI -1.03 to 1.22, and *P* = 0.87 > 0.05). Two studies analyzing PCO_2_ after complete paralysis or intubation preoxygenation showed that there was no difference between the two groups (MD 2.76, 95% CI -1.74 to 7.26, and *P* = 0.23 > 0.05).

**Conclusions:**

THRIVE does not have a greater advantage over a facemask in improving apnea time, oxygenation time, PCO_2_, and SpO_2_, but it has an advantage in improving arterial partial pressure of oxygen (PaO_2_) after preoxygenation, which can improve PaO_2_ well. This trial is registered with the protocol registration number CRD42021268143.

## 1. Introduction

Transnasal Humidified Rapid-Insufflation Ventilatory Exchange (THRIVE) is a noninvasive respiratory support technique that delivers continuous, warm, humidified 100% oxygen at high flow rates (up to 120 L/min) through a nasal cannula to clinically apneic patients, ensuring their relative oxygen concentration is constant [1]. With the development of oxygen therapy technology, it has received increasing attention and application. THRIVE has been used in intensive care units (ICU), respiratory medicine, and perioperative preoxygenation [2, 3].

Asphyxia oxygenation is the oxygenation of a patient in the absence of spontaneous breathing or mechanical ventilation [4]. The THRIVE technique is currently most widely used in the perioperative period; the effectiveness of THRIVE can facilitate the patency of the patient's upper airway, especially in preoperative preoxygenation [5, 6]. The purpose of the preoxygenation technique is to increase the body's oxygen stores, delay the body's consumption of oxygen during apnea, delay the onset of arterial hemoglobin desaturation, and delay the rapid decline in oxygenation [3]. Preoxygenation is required preoperatively to improve the safety of intubation in surgical patients because of the unpredictability of ventilation and intubation difficulties [7, 8]. THRIVE provides a high flow rate of gas that produces a continuous positive pressure effect on the airway [9], thus providing physical pressure support to the upper airway, which raises the oropharyngeal pressure, and the positive pressure generated by the high flow rate of oxygen, which reopens the atrophied alveoli and can promote alveolar reopening [10]. The technique has not yet received a large amount of clinical data to support its safety and effectiveness, and only a small sample of randomized controlled trials has been conducted to study it, which still lacks evidence. There is still controversy about THRIVE and facemask oxygenation, including two groups of studies for the duration of inspiratory pause after preoxygenation, PaO_2_ after preoxygenation, and SpO_2_ after successful intubation, as well as after waiting for the patient to be completely paralyzed by anesthesia or after preoxygenation by intubation [3, 11–14]. Therefore, we conducted a systematic review and meta-analysis to resolve the controversial points and provide favorable evidence-based medicine.

## 2. Methods

The protocol and guidance for this study were performed in accordance with Preferred Reporting Items for Systematic Reviews and Meta-Analyses (PRISMA) [15]. The protocol for this review was registered with PROSPERO (CRD42021268143).

### 2.1. Inclusion Criteria

The inclusion criteria include the following:

1. American Society of Anesthesiologists (ASA) grades to assess physical condition classes I~IV
2. Patients requiring tracheal intubation, facemasks, or noninvasive ventilation under anesthesia
3. Randomized controlled trial as the type of study

### 2.2. Exclusion Criteria

1. The exclusion criteria include the following:Body mass index (BMI) > 35 kg · m^−2^, as well as pregnant women and children
2. Nonrandomized controlled trials, animal studies, conference abstracts, case reports, reviews, and other nonclinical research literature as the types of studies
3. Literature with incomplete or unavailable valid data

### 2.3. Search Strategy

PubMed, Embase, Web of Science, and Cochrane Library have search restrictions from the first publication until 15 July 2021 for full-text articles in English. We searched for the keyword “Transnasal Humidified Rapid-Insufflation Ventilatory Exchange.” The relevant references in the literature were searched manually to avoid missing relevant studies. The remaining articles were filtered according to their titles and abstracts, and the papers thus selected were then reviewed in full.

### 2.4. Study Selection and Data Collection Process

Two investigators (LYK and YJZ) independently screened the literature, study selection, data extraction, quality of evidence, and risk of bias assessment based on the inclusion and exclusion criteria, and if a disagreement arose during the screening process, a third investigator was consulted to make a decision [16].

The extracted data include the following: study; research type; groups; cases; age (years), mean (SD); sex (male/female); BMI (kg/m^2^), median (IQR [range]); the median of PaO_2_ after preoxygenation, median (IQR [range]); time taken for intubation (s), median (IQR [range]); SpO_2_ after successful intubation, median (IQR [range]); apnea time (s), median (IQR [range]); and PCO_2_ after complete paralysis or intubation preoxygenation. A table was created using Microsoft Excel 2016 software (spreadsheet software, Microsoft Corporation) to extract and record the data from the literature.

### 2.5. Assessment of Risk of Bias and Quality of Evidence

The Cochrane Risk of Bias Assessment Tool (*Cochrane Reviewers' Handbook 4.2.2* and RevMan 5.4 software) was used to assess the quality of the included studies on 6 indicators, including random assignment scheme, allocation concealment, blinding, outcome data integrity, selective reporting bias, and other biases, judged on three criteria: low risk of bias, unclear risk of bias, and high risk of bias. In the statistical process, the quality assessment was categorized: 5 items and above were considered low risk of bias; 3 to 4 items were considered moderate risk of bias; and those with less than 3 items were considered high risk of bias [17].

### 2.6. Statistical Analysis

Statistical methods were performed using Stata 14.0 software (StataCorp LLC, 4905 Lakeway Drive, College Station, USA) and Review Manager analysis software (RevMan 5.4; Cochrane Collaboration, Oxford, UK) provided by the Cochrane Collaboration for meta-analysis. If the original study data were the median and interquartile range (IQR) or range representing continuous variables, the continuous variables were transformed into mean ± SD by calculating (*x* ± *s*) by the method proposed by Wan et al. [18]. The results were used as the mean difference (MD) or standardized mean difference (SMD), respectively, and their 95% confidence intervals (CI) were provided. The degree of variation among the results of the included studies was tested, and if *P* > 0.10 and *I*^2^ < 50%, a fixed-effects model was used; otherwise, the heterogeneity of the included literature will be looked at by subgroup analysis, sensitivity analysis, or metaregression. When heterogeneity cannot be explained by clinical issues or methods with the degree of the heterogeneity being within acceptable limits, a random-effects model could be used. The degree of asymmetry of the funnel plot was used to detect publication bias.

## 3. Results

### 3.1. Literature Search

**Figure 1:**
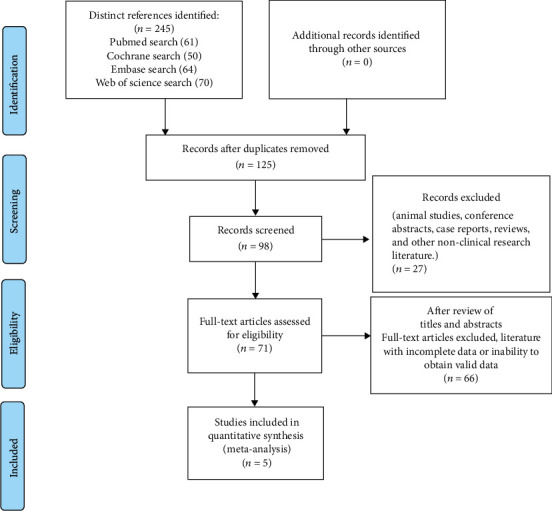
The Preferred Reporting Items for Systematic Reviews and Meta-Analyses outline a flow chart of retrieved, included, and excluded randomized controlled trials.

Our search initially identified 245 records. After removing duplicates, 125 studies remained. 27 studies from animal studies, conference abstracts, case reports, reviews, and other nonclinical research literature were excluded, leaving 71 studies. After reviewing titles and abstracts, 66 studies were excluded due to full-text articles, incomplete data, or unavailability of valid data. Five studies were eventually included (Figure 1), all of which were randomized controlled trials with a total of 235 patients included in the five articles, and the literature screening process and results are shown in Table 1.

### 3.2. Risk of Bias Assessment

**Figure 2:**
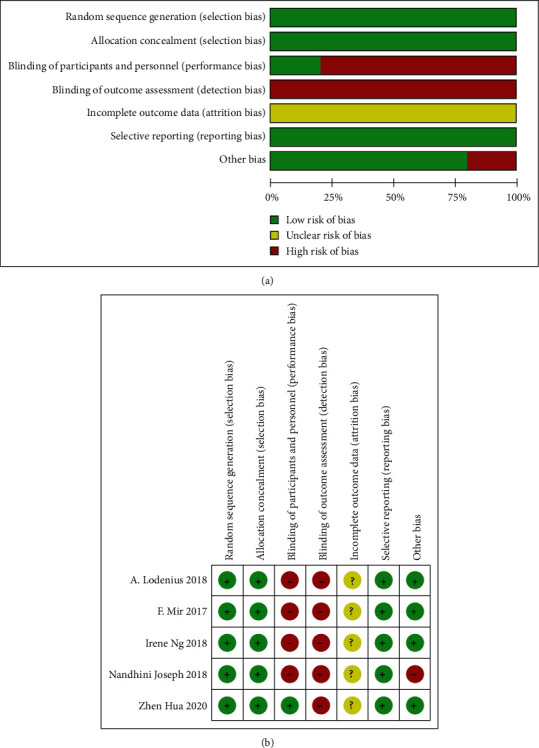
(a) The distribution of the methodological quality of included studies. (b) Methodological quality of included studies.

Most of the included studies were considered to have an unclear risk of bias. No information on allocation concealment was available, and only a few studies reported random sequence generation. One of the studies was a single-blind randomized control, and the others were open randomized controls. Detailed information on the risk of bias assessment is shown in Figure 2. Because of the limited number of included studies, there may be publication bias.

### 3.3. Study Characteristics

Five papers mainly pooled the effect of Transnasal Humidified Rapid-Insufflation Ventilatory Exchange (THRIVE) vs. facemask on improving apnea time, intubation time, PaO_2_, PCO_2_, and SpO_2_ after preoxygenation in anesthetized patients or ICU patients. A meta-analysis finally yielded one statistically significant indicator.

#### 3.3.1. Time Is Taken for Intubation

**Figure 3:**
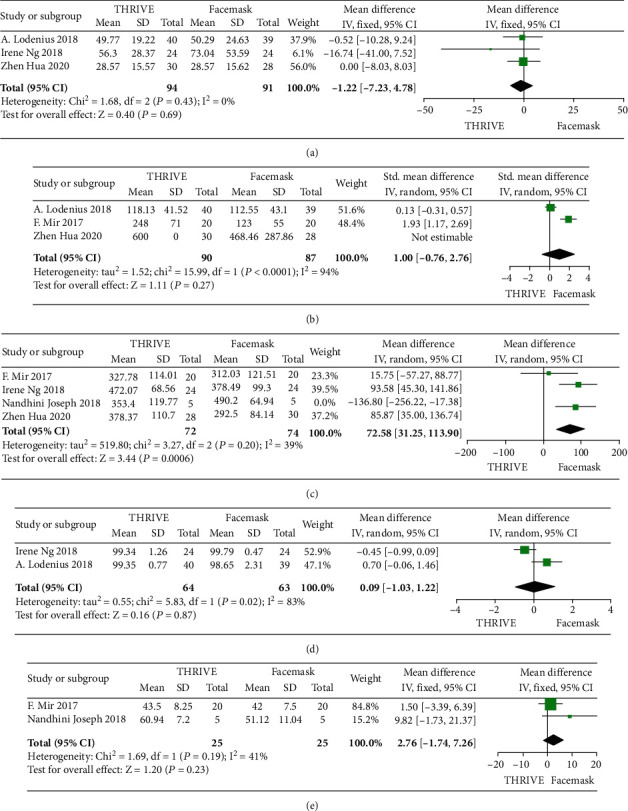
(a) Time is taken for intubation. (b) Apnea time. (c) PaO_2_ after preoxygenation. (d) SpO_2_ after successful intubation. (e) PCO_2_ after complete paralysis or intubation preoxygenation. For each trial, the square depicts the mean difference and the horizontal lines on either side of it represent the 95% CI. The summary result is presented as a diamond. IV: inverse variance.

Three literature studies were conducted for intubation time. The three pieces of literature of this study, tested for heterogeneity *I*^2^ = 0% < 50% and *P* = 0.43 > 0.1 for the *Q* test, suggest that there is no heterogeneity among the literature selected for this study and fixed effects can be selected for meta-analysis. To ensure the accuracy and stability of the study, sensitivity analysis was continued. Sensitivity analysis was performed on the three papers in this study, and none of them caused significant interference with the results of this meta-analysis, implying that this study has good stability. Three randomized controlled trials were conducted with intubation time as the primary observation. Analysis of both studies showed that the THRIVE group compared with the facemask group (MD -1.22, 95% CI -7.23 to 4.78, and *P* = 0.69 > 0.05; Figure 3(a)) suggested no difference in intubation time between the three groups. Although the THRIVE group was shown to be superior to the facemask group in terms of intubation time in one study, the final analysis resulted in no advantageous difference between the two groups. There was no significant effect on intubation time in the THRIVE group compared to the facemask group after the preoxygenation advantage.

#### 3.3.2. Apnea Time

For apnea time, three papers were analyzed, and after heterogeneity analysis, only one paper was not significant in the analysis. The study's three papers, after the heterogeneity test *I*^2^ = 94% > 50% and *P* = <0.001 for the *Q* test, suggest that there is heterogeneity between the literature selected in this study, and in the study of Hua et al., the difference of the documented mean and standard deviation and other two literature data is larger, so choose SMD to merge the effect and choose a random effect for meta-analysis. The three randomized controlled trials used apnea time as the primary observation. Analysis of these three studies showed that the THRIVE group compared with the facemask group (SMD 1, 95% CI -0.76 to 2.76, and *P* = 0.27 > 0.05; Figure 3(b)) suggested no difference in intubation time between the two groups, and the THRIVE group compared with the facemask group had no significant effect on apnea time after the preoxygenation advantage, and there was no difference in apnea time between the two groups.

#### 3.3.3. PaO_2_ after Preoxygenation

For PaO_2_ after preoxygenation, four papers were analyzed, and after heterogeneity analysis, one paper was discarded and the weight of this paper was adjusted to 0. The four papers of this study, tested for heterogeneity *I*^2^ = 80% > 50% and *P* = 0.002 < 0.1 for the *Q* test, suggest that heterogeneity exists among the papers for meta-analysis. To ensure the accuracy and stability of the study, sensitivity analysis was continued.

The sensitivity analysis of the four papers in this study found that the study of Joseph et al. had a large effect on heterogeneity, and the results of the heterogeneity test after removing the study showed that there was no heterogeneity in the remaining three papers (*I*^2^ = 39% < 50%, *P* = 0.20 > 0.1), and considering the small sample size of the study and the short preoxygenation time, the study had better stability after exclusion. A random-effects meta-analysis was performed, and the details are shown in the following figure. The three randomized controlled trials used PaO_2_ after preoxygenation as the primary observation. Analysis of the three studies showed that the THRIVE group compared with the facemask group (MD 72.58, 95% CI 31.25 to 113.90, *Z* = 3.44, and *P* < 0.001; Figure 3(c)) suggested that the PaO_2_ after preoxygenation was statistically significant in both groups, with the THRIVE group producing higher PaO_2_ after preoxygenation compared with the facemask group, making it superior to the facemask.

Bias tests and sensitivity analyses were conducted, and three papers were located near the midline by sensitivity analysis. Three papers have good stability (Figure 3(c)). So it can be judged that there is no publication bias in the literature of this study.

There was a significant difference in PaO_2_ between the THRIVE group and the facemask group in patients with apnea time up to 1-10 minutes. Increasing the PaO_2_ of patients during apnea directly affects patients' tolerance during apnea, provides relatively longer oxygen demand in preoperative preparation or critically ill patients, indirectly prolongs the time of oxygen consumption in brain cells, and provides supply for brain oxygen demand. The THRIVE group was superior to the facemask group in PaO_2_ after preoxygenation.

#### 3.3.4. SpO_2_ after Successful Intubation

For SpO_2_ after successful intubation, two papers were analyzed. The two papers of this study, tested for heterogeneity *I*^2^ = 82.3% > 50% and *P* = 0.017 > 0.1 for the *Q* test, suggest that heterogeneity exists between the literature selected for this study and that random effects can be selected for meta-analysis. To ensure the accuracy and stability of the study, the sensitivity analysis was continued. The two randomized controlled trials used the oxygen saturation SpO_2_ after successful intubation as the primary observation. Analysis of both studies showed that the THRIVE group compared with the facemask group (MD 0.09, 95% CI -1.03 to 1.22, and *P* = 0.87 > 0.05; Figure 3(d)) suggested that there was no statistically significant difference in oxygen saturation SpO_2_ after successful intubation between the two groups, and the THRIVE group compared with the facemask group in terms of oxygen saturation SpO_2_ after successful intubation suggested that there was no difference in SpO_2_.

No significant differences in oxygen saturation were found between the THRIVE and facemask groups in SpO_2_ analysis after preoxygenation and after apnea. Mean oxygen saturation was over 90% in both the THRIVE and facemask groups. It is important to note that the cutoff time for apnea was set at 10 minutes to avoid complications of hypoxia and hypercapnia.

#### 3.3.5. PCO_2_ after Complete Paralysis or Intubation Preoxygenation

For PCO_2_ after complete paralysis or intubation preoxygenation, two papers were analyzed. Two randomized controlled trials used PCO_2_ after complete paralysis or intubation preoxygenation as the primary observation. Analysis of the two studies showed that the THRIVE group compared with the facemask group (MD 2.76, 95% CI -1.74 to 7.26, and *P* = 0.23 > 0.05; Figure 3(e)) suggested that there was no statistically significant difference in PCO_2_ after complete paralysis or intubation preoxygenation between the two groups. Oxygenation was not statistically significant, and there was no difference in PCO_2_ after complete paralysis or intubation preoxygenation in the THRIVE group compared with the facemask group.

In our study, the reason why no significant difference in PaCO_2_ was found between the THRIVE and facemask groups may be related to reduced pulmonary compliance and increased pulmonary atelectasis.

## 4. Discussion

Our meta-analysis suggests that Transnasal Humidified Rapid-Insufflation Ventilatory Exchange (THRIVE) is receiving increasing attention and application as an effective noninvasive mode of ventilation in the treatment of clinical anesthesia and critically ill patients, but the conclusions reached in clinical studies of THRIVE are still controversial, and for the present study, THRIVE improves PaO_2_ in asphyxiated oxygenated patients, directly affects patients' apnea tolerance, provides relatively long oxygen consumption for anesthetized or critically ill patients, and improves the safety of tracheal intubation. Transnasal Humidified Rapid-Insufflation Ventilatory Exchange may be a better choice for preoxygenation.

In the five included articles, randomized controlled trials (RCTs) were used to compare apnea time, intubation time, PaO_2_, PCO_2_, and SpO_2_ after preoxygenation when patients were treated with THRIVE and facemask preoxygenation. The ultimate goal of this study was to determine which study had the greatest benefits for patients with asphyxia. The final purpose of the study is the same, but the research baseline is still insufficient. For patients who are about to be intubated, there are many mixed factors. For patients with respiratory diseases or other situations, the probability of general tracheal intubation will be large, and the overall health of patients needs to be comprehensively evaluated. Therefore, whether patients need tracheal intubation should be carefully judged. The following reasons are urgent or necessary for tracheal intubation. (1) Patients need general anesthesia. (2) Rescue critical patients and patients with respiratory and cardiac arrest. (3) When the patient's breathing cannot maintain the body's needs or cough reflex gradually weakened patients, nonsurgical patients need to rely on endotracheal intubation to maintain the patient's oxygenation. (4) Acute respiratory failure caused by any reason requires urgent intubation.

One study showed that compared to facemasks [19], THRIVE had no significant effect on the incidence of hypoxemia (venous oxygen saturation (SpO_2_) < 80%), time to asphyxia, arterial partial pressure of oxygen (PaO_2_) after preoxygenation, and PaO_2_ and PaCO_2_ after intubation, and there were no statistically significant differences in 28-day morbidity and mortality, serious complications (significant hypotension, use of antihypertensive drugs, or cardiac arrest), or length of ICU stay. In a multicenter RCT in 2019 [20], the incidence of severe hypoxia with preoxygenation with noninvasive ventilation was lower than that with THRIVE in patients with acute hypoxic respiratory failure (oxygenation index ≤ 200 mmHg). In a randomized double-blind study, preoxygenation with THRIVE at 60 L/min combined with noninvasive ventilation (inspiratory pressure 10 cm H_2_O, expiratory pressure 5 cm H_2_O, FiO_2_ 100%) for preoxygenation improved oxygenation during intubation and reduced the incidence of severe hypoxia (SpO_2_ < 80%) compared with noninvasive ventilation alone.

In this study, the meta-analysis method was used to control the baseline of patients. According to the inclusion and exclusion criteria, the articles that can be analyzed were finally found. The results showed that although there was still heterogeneity in some articles in the analysis process, the articles with large heterogeneity were eliminated from the study by statistical methods to reduce the heterogeneity of the study. The results showed that the PaO_2_ concentration in the THRIVE group after preoxygenation was significantly better than that in the facemask group, which showed that the THRIVE group had advantages in asphyxia oxygenation. And there was no significant difference between the two groups in the apnea time, oxygenation time, PaO_2_, PCO_2_, and SpO_2_ after preoxygenation. Therefore, THRIVE does not have a greater advantage over a facemask in improving apnea time, oxygenation time, PCO_2_, and SpO_2_, but it has an advantage in improving arterial partial pressure of oxygen (PaO_2_) after preoxygenation, which can improve PaO_2_ well. This study believed that Transnasal Humidified Rapid-Insufflation Ventilatory Exchange still had potential advantages in patients' oxygenation.

In clinical practice, due to the uniqueness of the study, given the small number of studies included in the literature and the small sample size of the study, it was not possible to avoid detection bias, resulting in these results remaining controversial, probably as a result of the limited sample size, which is a limitation of the systematic review. Further studies with large sample sizes are urgently needed to explore this issue. In this study, further large sample size studies and more clinical studies are needed to examine the safety and efficacy of THRIVE so that THRIVE can be more widely used, safer, and more effective in the treatment of clinical anesthesia and critically ill patients.

## Figures and Tables

**Table 1:** Study characteristics and outcomes of interest assessed in included studies.

Study	Research type	Groups	Cases	Age (years), mean (SD)	Sex (male/female)	BMI (kg/m^2^), median (IQR [range])	The median of PaO_2_ after preoxygenation (mmHg/kPa), median (IQR [range])	Time taken for intubation (s), median (IQR [range])	SpO_2_ after successful intubation, median (IQR [range])	Apnea time (s), median (IQR [range])	PCO_2_ after complete paralysis or intubation preoxygenation (mmHg/kPa)
Ng et al. [14]	Prospective randomized controlled trial	THRIVE	24	52.6 (17.4)	13/11	26.6 (4.3)	471 (IQR 429–516 [range 185–550])	52 (40–76 [29–315])	99.7% (98.4%–100% [91.6%–100%])	NA	NA
		Facemask	24	57.3 (15.6)	13/11	27.4 (3.5)	357 (interquartile range (IQR) 324–450 [range 183–550])	58 (45–113 [30–220])	100% (99.4%–100% [94.5%–100%])	NA	NA
Hua et al. [11]	Randomized controlled trial	THRIVE	30	73.10 ± 5.05	16/14	24.72 ± 3.03	378.37 (110.70)	25 (20~40)	NA	600 (600–600)	NA
		Facemask	28	70.64 ± 4.28	17/11	25.03 ± 5.55	292.50 (84.14)	25 (20~40)	NA	600 (231.5–600)	NA
Lodenius et al. [13]	Prospective randomized nonblinded clinical trial	THRIVE	40	55.6 (17.3)	20/20	24.5 (4.6)	NA	48 (38–63 [10–146])	99% (99–100 [96–100]%)	116 (92–146 [63–249])	NA
		Facemask	39	51.8 (19.6)	16/23	26.0 (5.1)	NA	51 (34–66 [22–261])	99% (97–100 [70–100]%)	109 (86–142 [37–291])	NA
Mir et al. [3]	Randomized controlled trial	THRIVE	20	46.4 (16.8)	11/9	26 (24.5–31.5 [22–46])	43.7 (15.2)	NA	NA	248 (71)	5.8 (1.1)
		Facemask	20	51.8 (21)	9/11	25 (23–29.25 [21–48])	41.9 (16.2)	NA	NA	123 (55)	5.6 (1.0)kPa
Joseph et al. [12]	Randomized controlled trial	THRIVE	5	49.80 ± 26.82	3/2	NA	353.4 ± 119.77	NA	NA	NA	60.94 ± 7.20
		Facemask	5	57.80 ± 10.80	2/3	NA	490.20 ± 64.94	NA	NA	NA	51.12 ± 11.04

Patient characteristics. Data are presented as mean (SD), number (proportion), or median (interquartile range). BMI: body mass index; SD: standard deviation; IQR: interquartile range; THRIVE: Transnasal Humidified Rapid-Insufflation Ventilatory Exchange.

## Data Availability

All data relevant to the study are included in the article or uploaded as supplementary information. All data can be obtained from the included studies.

## Supplementary Materials

Supplementary Materials The PRISMA Group (2020). Preferred Reporting Items for Systematic Reviews and Meta-Analyses.
